# Altered Co-Expression Patterns of Mitochondrial NADH-Dehydrogenase Genes in the Prefrontal Cortex of Rodent ADHD Models

**DOI:** 10.3390/ijms262211079

**Published:** 2025-11-16

**Authors:** Polina A. Sylko, Arina A. Gromova, Zoia S. Fesenko, Evgeny V. Kanov, Anna B. Volnova, Raul R. Gainetdinov, Anastasia N. Vaganova

**Affiliations:** 1 Institute of Translational Biomedicine, St. Petersburg State University, Universitetskaya Nab. 7/9, 199034 St. Petersburg, Russia; 2 Center for Transgenesis and Genome Editing, St. Petersburg State University, Universitetskaya Nab. 7/9, 199034 St. Petersburg, Russia

**Keywords:** ADHD, hyperactivity, prefrontal cortex, mitochondria, mitochondrial DNA, transcriptomic data

## Abstract

Altered mitochondrial function is implicated in disorders characterized by prefrontal cortex activation deficits, including attention deficit hyperactivity disorder (ADHD). The expression of mitochondrial DNA-coded respiratory chain complex I genes (*ND1–ND6*) in the prefrontal cortex of ADHD animal models was estimated in the present study. ND gene expression was assessed in two publicly available datasets: GSE117357 (*Adgrl3* knockout mice) and GSE173926 (*MYT1L* heterozygous knockout mice). Additionally, we measured *NDs* gene expression via qPCR in dopamine transporter knockout (*DAT*-KO) rats and their heterozygous (*DAT*-Het) littermates. Transcriptomic analysis revealed consistent *ND1–ND6* expression profiles across both datasets, and co-expression among *ND* genes was significantly enhanced in ADHD models compared to wild-type controls. Whole-transcriptome analysis identified associations between *ND3* and *ND4L* expression and genes involved in neural tissue-specific processes, exclusively in ADHD models. In *DAT*-KO and *DAT*-Het rats, *NDs* gene co-expression increased. Furthermore, in *DAT*-Het rats, which do not exhibit hyperactivity, the upregulation of *ND4L* expression relative to wild-type littermates was demonstrated. The observed changes in mitochondrial complex I gene co-expression in ADHD models suggest mitochondria may serve as a prospective target for adjuvant therapy. These findings highlight the need for further investigation into mitochondrial contributions to ADHD pathophysiology.

## 1. Introduction

Attention deficit hyperactivity disorder (ADHD) is a neurodevelopmental disorder characterized by early-onset symptoms, including hyperactivity, impulsivity, and inattention [1]. The disorder is also associated with deficits in executive functions such as problem-solving, vigilance, inhibitory control, and working memory, as well as disruptions in language processing and sleep patterns. Longitudinal studies link ADHD to increased risks of comorbid conditions, including substance use disorders, mood and personality disorders, suicidal behavior, and eating disorders [2,3].

Mitochondrial dysfunction is considered a possible contributing factor to the etiology of ADHD [4]. ADHD patients had higher mitochondrial DNA (mtDNA) copy numbers [1,4,5] and sporadic mtDNA mutations [5]. Also, altered expression of genes involved in mitochondrial functioning occurs in the prefrontal cortex (PFC) of ADHD model rats (spontaneously hypertensive rats) [4,5]. mtDNA haplogroups B4 and D4b (only in girls) are associated with developing ADHD symptoms. mtDNA haplogroup B5 was a protective factor for ADHD in Korean children [5,6]. Meanwhile, mtDNA copies may decrease with the reduction in ADHD severity in patients receiving treatment [5].

Mitochondria are complex endosymbionts that evolved from purple nonsulfur bacteria [7]. These organelles implement energy production through oxidative phosphorylation (OXPHOS) and simultaneously are involved in calcium storage, reactive oxygen species production and elimination, maintenance of redox homeostasis, and apoptosis [6,8,9]. In the nervous system, mitochondrial buffering of cytoplasmic Ca^2+^ is important for maintaining synaptic transmission [10] and vesicle exocytosis [11,12]. The mitochondria can also take up neurotransmitter dopamine (DA) and metabolize it, producing reactive oxygen species (ROS) [13]. Higher levels of DA are toxic to mitochondria and impair ATP production [14,15,16,17]. Astrocytic metabolism of gamma-aminobutyric acid (GABA) by mitochondrial GABA transaminase is vital for normal CNS functioning [18].

Each cell contains up to 1000 copies of the mitochondrial genome, mtDNA. In contrast to nuclear DNA, mtDNA is prone to damage because of constant exposure to ROS [8], which are byproducts of the electron transport chain [12]. The human mtDNA encodes for 13 components of the mitochondrial respiratory chain, including the components of the membrane Complex I (NADH:ubiquinone oxidoreductase) domain ND1–ND6 [13]. Complex I is the entrance point of electrons to the respiratory chain. The domain in the inner mitochondrial membrane is involved in proton translocation; the other domain protrudes into the mitochondrial matrix and is responsible for oxidation of NADH [16].

Complex I is implicated in the pathophysiology of neuropsychiatric diseases [7,14,19]. The total mtDNA deletions [20] and decreased amount of complex I protein level in the dorso-lateral prefrontal cortex in schizophrenia, bipolar disorder [14], major depressive disorder [8,12,21,22,23], and autistic spectrum disorder [6,11,16,24] subjects lead to elevated ROS and predispose neuronal cells to mitochondrial-dependent apoptosis [16,25,26]. Extracellular vesicles derived from neurons demonstrate decreased mRNA levels of complexes I and III in schizophrenic patients [27]. Expression of Complex I subunits ND1, ND2, ND4, ND4L, and ND5 is deregulated in animal models of various conditions [28]. For example, expression of ND4 and ND6 mRNA was suppressed in the nucleus accumbens and striatum after nicotine treatment [29], as well as ND2 mRNA expression being suppressed in the striatum and midbrain in a Parkinson’s disease rat model [30], or in the rat hippocampus, cortex, and cerebellum after X-ray irradiation [31]. Additionally, it was demonstrated that acute immobilization stress reduces the expression of ND1, ND3, and ND6 mRNA in the rat hippocampus [32].

The availability of postmortem brain samples of ADHD patients is limited [33,34], and the published human studies for the molecular background of ADHD are often based on peripheral tissue samples or blood [1,4]. In this context, animal ADHD models, or models of symptoms similar to ADHD, are valuable to study different aspects of ADHD etiology and pathogenesis [35]. Hence, the aim of the present study is the evaluation of the deregulation of expression and functional associations of complex I subunits genes, which are coded by mtDNA in the PFC, i.e., *ND1–6*, in ADHD rodent models. We reviewed public transcriptomic datasets from the Gene Expression Omnibus (GEO) database [36] and compared the received data with *ND1–6* expression in rats with dopamine transporter (DAT) gene knockout. *DAT* knockout (*DAT*-KO) results in hyperactivity, repetitive non-goal-directed behavior, and sensory gating deficits [37], accompanied by impaired learning ability and ability of information processing and transmission [38,39,40,41]. Upon exposure to a novel environment, *DAT*-KO rodents exhibit persistently elevated spontaneous locomotor activity [42,43], failing to habituate even after 240 min, a stark contrast to wild-type (WT) mice, which typically show active exploration for only 30–40 min. Moreover, repeated exposure to an open field does not induce habituation in *DAT*-KO animals [44]. Notably, pharmacological blockade of both D1 and D2 dopamine receptors effectively attenuates this hyperactivity [45], underscoring the dopamine-dependent nature of these behavioral alterations. Because of these impairments, *DAT*-KO animals are considered the models of ADHD [46,47,48]. In parallel, we studied heterozygous *DAT*-Het knockouts, which did not demonstrate significant behavioral abnormalities, including hyperactivity, inattention, and increased stereotypical and perseverative behaviors [49], except impairments in social interaction within groups [50], as well as deficits in maternal behavior and disturbances to the sleep–wake cycle [51]. *DAT*-Het rats show increased motor activity, crossing more squares in the open field and elevated plus maze tests. In the extrapolation escape test, they exhibited an increased latency to dive and made more unsuccessful avoidance attempts [52]. Overall, *DAT*-Het rats exhibit an intermediate pattern of behavioral impairments that are milder than those observed in *DAT*-KO knockout animals. Therefore, rats with one copy of the *DAT* gene may be a more accurate model for human disorders associated with disturbances in dopaminergic transmission.

Several genetic polymorphisms, including single nucleotide polymorphisms (SNP), identified in genes active in the PFC and other brain areas, are associated with attention deficit hyperactivity disorder [53,54]. Among these, mitochondria-related gene polymorphisms have also been identified, including those in the nuclear-encoded gene for the Complex I subunit NDUFAF2 [55] and the mitochondrial gene *ND2* [56].

In different tissues that differ in energy demands, the level of mtDNA expression may vary and is regulated by distinct mechanisms [19,20]. For example, the deregulation of mtDNA expression under damaging conditions varies depending on the tissue type, as demonstrated in mitochondrial transcription factor A (TFAM)-overexpressing mice. In these mice, mtDNA gene expression was more severely disrupted in skeletal muscle compared to heart muscle [20]. Additionally, Complex I activity and expression are reduced in cell lines derived from schizophrenic patients and prefrontal cortex samples [20]. Moreover, Complex I activity and expression are decreased in schizophrenic patient-derived cell lines and prefrontal cortex samples [14,20], but increased in platelets [16]. These discrepancies highlight the limitations of using low-invasive sampling methods to study tissue-specific mtDNA expression. Thus, we assessed the expression of *ND1–ND6* mRNAs as well as ATP production in the PFC in this model.

## 2. Results

### 2.1. Data Search and Inclusion

**Figure 1:**
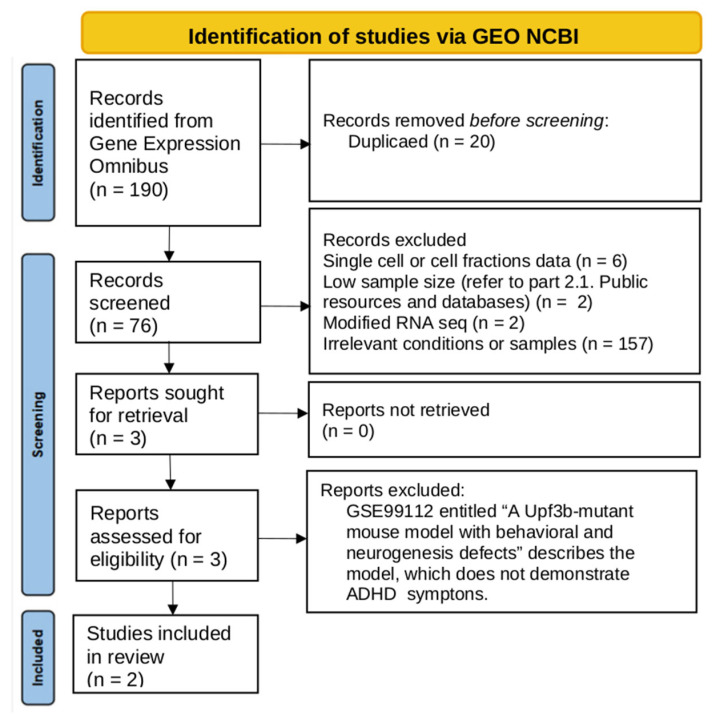
Flowchart of the data search.

From an initial search of the GEO database, we identified 190 records. After the exclusion of non-relevant datasets, three reports were included for final identification (Figure 1). The GSE99112 was excluded because the *Upf3b*-mutant mouse did not demonstrate hyperlocomotion and seems to be a model for the other neurodevelopmental disorders rather than ADHD [57]. Two RNA-seq datasets listed in Table 1 were included in the comparative analysis.

### 2.2. ND1–6 Expression in the Prefrontal Cortex in Mouse Models of ADHD RNA-Seq Data

**Figure 2:**
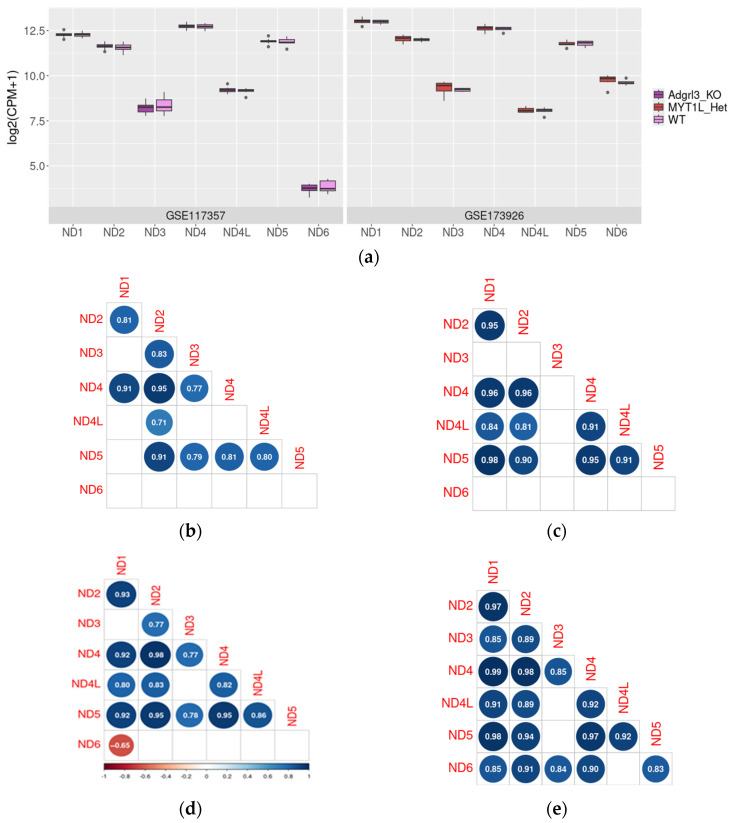
The *NDs* mRNA expression level in the PFC in wild-type mice and mice with mutations associated with ADHD-like behavior (in GSE117357 and GSE173926); the upper, middle, and lower lines of boxplots represent first quartiles, medians, and third quartiles (**a**). Correlations between *NDs* mRNA expression levels in the wild-type mouse PFC (**b**,**d**) for GSE117357 and GSE173926, respectively), PFC of *Adgrl3*-KO mice in the GSE117357 dataset (**c**), and *MYT1L* heterozygous knockout in GSE173926 (**e**). For *Adgrl3*-KO mice and their WT littermates, *n* = 6 for each group; for *MYT1L* heterozygous knockout mice and their WT littermates, *n* = 10 for each group.

*ND1–6* expression level distribution in PFC samples was congruent in two datasets included in the analysis, except for *ND6* mRNA levels, which are more than ten times higher in GSE173926 (828.1 ± 138.92) compared to GSE117357 (13.2 ± 2.78). No significant changes in *ND1–6* mRNA expression levels were identified in both ADHD models (*p* > 0.05, Figure 2a).

Meanwhile, when correlations between *ND* genes mRNA expression levels were estimated in WT mice and mice with genetic defects associated with ADHD-like symptoms were compared, it was revealed that in model animals, *NDs* mRNAs co-expression patterns are more complex and include more statistically significant (*p* < 0.05) correlations between the different *NDs* mRNA levels than wild-type samples (Figure 2b–d).

### 2.3. ND1–6 Expression in the Prefrontal Cortex in Rat DAT-KO ADHD Model

**Figure 3:**
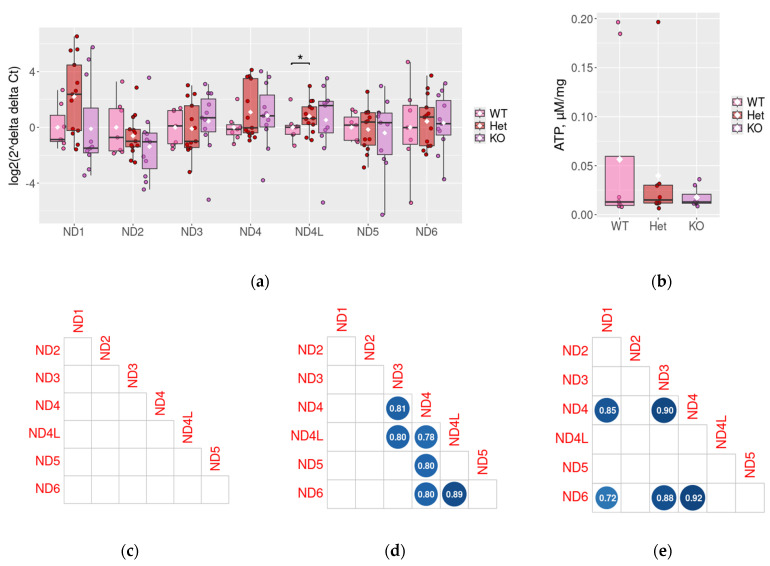
Expression of *ND* genes and ATP concentration in D in rat lines lacking the dopamine transporter PFC: qPCR results in WT, *DAT*-Het, and *DAT*-KO rats. Each dot represents expression level in a sample, and expression levels of genes were normalized to the expression of the housekeeping gene GSPDH by the 2^−ΔΔCt^ method; white diamonds indicate mean values; *—*p* < 0.05 (**a**), normalized ATP levels in WT, *DAT*-Het, and *DAT*-KO rats’ PFC (**b**), and correlations between gene expression (in Ct) in WT (**c**), *DAT*-KO heterozygous (**d**), and *DAT*-KO rats (**e**). Only statistically significant Pearson’s correlations were represented. No significant correlations were identified in the WT samples. Homozygous DAT knockout (*DAT*-KO, *n* = 11), heterozygous DAT knockout (*DAT*-Het, *n* = 13), and wild-type controls (WT, *n* = 6).

Considering the pronounced increase in ND gene expression level correlation in mouse ADHD models’ PFC samples, we evaluated the expression pattern of these genes in the PFC of *DAT*-Het and *DAT*-KO rats. We identified the ~2-fold change in ND4L expression level in *DAT*-Het rats, which are not hyperactive and demonstrate impaired social behavior and slight hyperlocomotion, but not pronounced hyperactivity [43] (*p* < 0.05, Figure 3a). Meanwhile, the comparison of *DAT*-KO rats and WT littermates did not reveal significant differences in *ND* genes expression levels.

No significant correlations were identified between *ND* genes in WT PFC (Figure 3c). However, in both *DAT*-Het and *DAT*-KO animals, statistically significant correlations were revealed for *ND3* and *ND4* gene expression and *ND4* and *ND6* gene expression. Additionally, some genotype-specific co-expressed gene pairs were revealed both in *DAT*-Het (Figure 3d) and *DAT*-KO (Figure 3e) rats.

### 2.4. Measurement of ATP Levels in DAT-Het and DAT-KO Rats

Taking into account that complex I is the entrance point of electrons to the respiratory chain for ATP biosynthesis [16], we measured the ATP concentrations in PFC samples harvested from *DAT*-Het and *DAT*-KO rats. Despite the identified differences in *ND* genes expression between WT and *DAT*-Het and *DAT*-KO rats, the ATP levels were not significantly changed in these animals. The identified tendency for ATP to decrease in *DAT*-deficient rats does not reach statistical significance (Figure 3b).

### 2.5. ND Genes Co-Expression Profiles in Mouse ADHD Models

Owing to the identified complexification of ND genes co-expression in mice carrying ADHD-like behavior-associated mutations, we hypothesized that similar alterations might affect interactions between ND genes and other genes involved in OXPHOS or related biological processes. To test this, we analyzed genes whose expression levels correlated with each ND gene in the GSE117357 and GSE173926 datasets. Genes were selected by Pearson’s correlation coefficient applying cut-off values of r > 0.75 and *p* < 0.05 for both datasets, considering the probability of this correlation level occurring by chance is very low [58,59]. The top 100 genes were selected for the analysis for each gene in each study group (the lists of genes that demonstrates the strongest co-expression with ND1–ND6 based on Pearson correlation are represented in Supplementary File S1). If fewer than 100 genes met these criteria, only those genes were included in the analysis.

**Figure 4:**
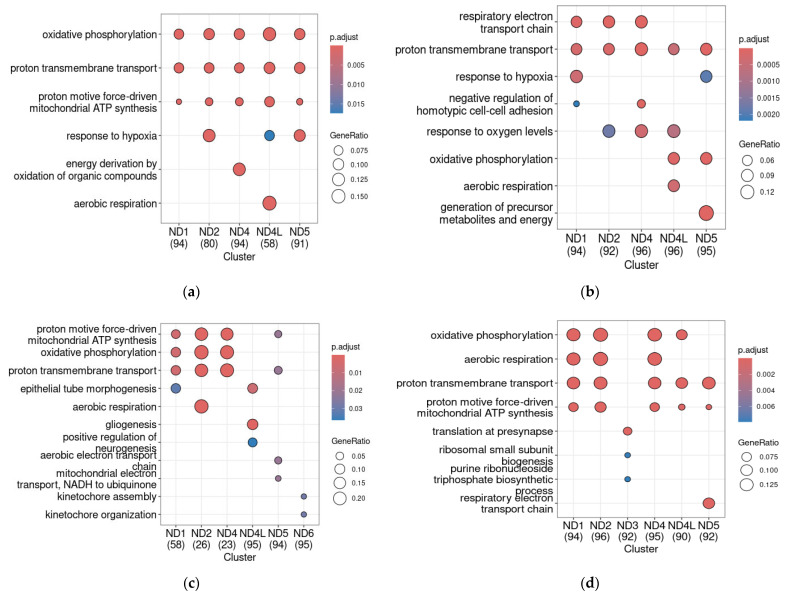
GO biologic process term enrichment for the top 100 genes co-expressed with *NDs* in the PFC in control WT mice in GSE117357 (**a**) and GSE173926 (**b**), *Adgrl3*-KO mice in the GSE117357 dataset (**c**), and *MYT1L* heterozygous knockout (**d**) (GSE173926). The number below the gene name represents the number of genes in each co-expressed gene cluster involved in the analysis after the transformation of Ensembl gene IDs to Entrez IDs (the gene lists are detailed in Supplementary File S1). The color gradient represents adjusted *p*-values, and the point size corresponds to the enrichment gene ratio.

In WT PFC samples, *ND* genes were predominately co-expressed with genes involved in proton transmembrane transfer, ATP synthesis, and other OXPHOS-related processes. This co-expression pattern is congruently reproduced in both datasets, despite technical differences and some fluctuations (Figure 4a,b and Supplementary Figure S1A,B which is an extended version of Figure 4a,b).

Meanwhile, in both the *Adgrl3*-KO mice in the GSE117357 dataset and the *MYT1L* heterozygous knockout in GSE173926, the top 100 gene clusters co-expressed with *NDs* include genes that are involved in the processes that are not related to energetic metabolism. In *Adgrl3*-KO mice in the GSE117357, these processes include neurogenesis and gliogenesis in the *ND4L*-co-expressed genes cluster and kinetochore organization in the *ND6* co-expressed gene cluster (Figure 4c and its extended version in Supplementary Figure S1C). In the *MYT1L*-Het (GSE173926), *ND3* is co-expressed with genes involved in the synaptic function (i.e., transport in presynapse, Figure 4d and its extended version in Supplementary Figure S1D). Also, in *Adgrl3*-KO mice, we revealed a pronounced loss of co-expression with other genes for *ND1*, *ND2*, and *ND4* (only 66, 26, and 23 genes met the selected criteria). On the other hand, the number of *ND4L*-co-expressed genes increased in this ADHD model (Figure 4c; the data for the number of genes, which are co-expressed with each *ND* gene based on Pearson’s correlation, are detailed in Supplementary File S2).

## 3. Discussion

**Figure 5:**
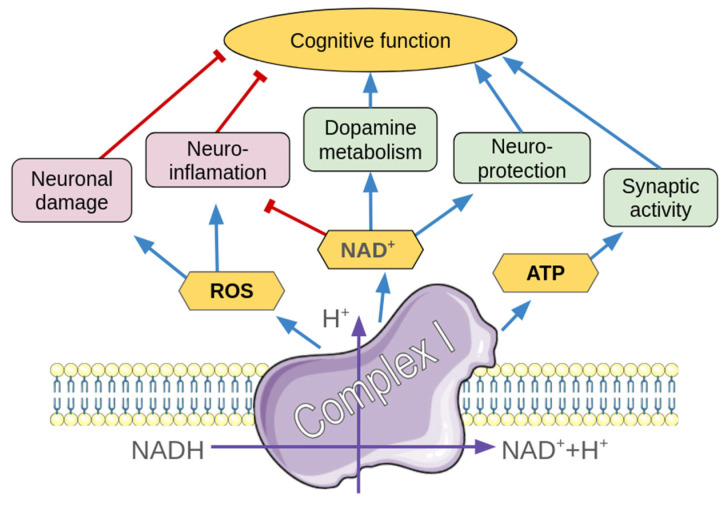
Diagram of the complex I role in regulation of processes involved in ADHD pathogenesis. Positive associations are marked by blue arrows, and negative regulation is indicated by red lines. Physico-chemical processes on Complex I are signed by purple arrows. ROS—reactive oxygen species, NAD—nicotinamide adenine dinucleotide, ATP—adenosine triphosphate. Parts of the figure were drawn by using pictures from Servier Medical Art. Servier Medical Art by Servier is licensed under a Creative Commons Attribution 3.0 Unported License.

Currently, there is a growing body of evidence for both altered mitochondrial function [60,61] and mtDNA polymorphism involvement in the development of ADHD [62]. In the present study, we were concentrated on the complex I subunits mitochondrial genes (*ND1–6*), which make up 7 of 13 protein-coding mitochondrial genes. In contrast to the bacterial genome, the mitochondrial genome does not consist of operons and is transcribed in two polycistronic precursor RNAs in opposite directions [63]. The *ND1–5* genes are located on a heavy strand of mtDNA, and the *ND6* gene is coded by the opposite light strand [64]. Complex I is the first enzyme of the respiratory chain, which is also responsible for the ROS generation and apoptosis regulation [26,65]. The involvement of Complex I, including the downstream ATP-synthesis pathway is schematically summarized in Figure 5. This enzyme includes both mtDNA-coded proteins and subunits, which are coded by the nuclear genome [65]. However, mtDNA is more prone to damage by ROS than the nuclear genome [65,66]. Considering the lifelong decrease in the PFC activation and volume in ADHD patients [67,68], we studied the expression of complex I mitochondrial genes in the PFC in rodent ADHD models.

Firstly, we assessed the *ND1–6* mRNA expression in available public transcriptomic datasets. Despite *ND1–5* being co-transcribed in polycistronic precursor RNA [63], the identified levels of these transcripts vary, and these variations are congruent in two datasets. The most expressed *ND* genes are *ND1*, followed by *ND2*, *ND4*, and *ND5*. The identified expression levels of *ND3* and *ND4L* genes are several times lower in both datasets (Figure 2a). However, coding sequences of *ND4* and *ND4L* overlap, so RNAseq results for these genes may be prone to bias [64]. At the same time, diverse proteins are required in the processing and maintenance of different *ND* mRNAs [65]. The *ND6* mRNA precursor is transcribed in another polycistronic RNA functionally independent from the heavy strand [65], and its lower expression compared to other *ND* mRNAs was described in brain and other tissues previously [69]. The discrepancy in levels of different mitochondrial gene transcripts in tissue samples despite the polycistronic transcription was described previously [63,69].

Previously, mitochondrial gene deregulation was identified in psychiatric conditions. In particular, a decrease in ND1, *ND2*, *ND3*, *ND4*, *ND5*, and *ND6* was identified in brain samples of schizophrenia or bipolar disorder patients [20,70,71], as well as in suicide victims [72] in several studies. Oppositely, in an anxiety-like behavior induced by multimodal chronic restraint stress in mice, the elevation of *ND1*, *ND2*, *ND4*, *ND5*, and *ND6* expression was revealed in the PFC of stressed animals [73]. Meanwhile, in other papers, the expression of all *ND* genes in PFC samples from patients with bipolar disorder, schizophrenia, or major depressive disorder does not significantly differ from control subjects [74]. For less severe disorders, like ADHD, the data are limited. In our study, we evaluated *ND1–6* expression in three rodent models of ADHD and did not reveal any significant changes in the expression levels of these genes. The observed changes in co-expression patterns may reflect reorganization of mitochondrial regulatory processes, potentially linked to mitochondrial regulatory systems damage or some compensatory adaptations in the pathologic conditions. Interestingly, the increased *ND4L* expression was revealed in PFC samples of *DAT*-Het rats. These animals are not hyperactive and are included in the study as an additional group that suffers from decreased DA reuptake in brain tissues, which is less pronounced than in *DAT*-KO rats [37]. Previously, *ND4L* mRNA increase was identified in an autistic spectrum disorder model in mice after the supplementation with L-proline, which ameliorated the behavior of these animals [75], so this molecular feature may be associated with some compensatory mechanism, but this suggestion needs further studies. The changes, identified in *DAT*-KO and *DAT*-Het rats, however, did not significantly affect the final ATP production. Meanwhile, other effects of these changes, like ROS production, were not investigated in the present study.

Despite the absence of changes in *ND* genes expression levels in all three ADHD models included in the analysis, the consistent increase in co-expression frequency and strength of co-expression between these genes was identified in the PFC samples of hyperactive animals with different mutations. Though the products of *ND* mitochondrial genes comprise one enzyme along with 38 nuclear genes of the complex I subunits [76], their expression regulation is complex, independent, and varies between tissues [77]. Mitochondria maintain higher levels of their genomic transcripts, and mRNA of nuclear genes expression is significantly low compared to mitochondrial genes but may be faster regulated if OXPHOS biogenesis needs to be modulated. On the other hand, nuclear and mitochondrial OXPHOS transcription programs are coregulated by mitochondrial-nuclear signaling pathways, and the mitochondrial genes expression is regulated by nuclear-encoded factors that are imported into mitochondria [78]. This may explain that *ND* genes are co-expressed with nuclear genes involved in the OXPHOS rather than among themselves.

In *Adgrl3* knockout, *ND4L* becomes co-expressed with genes involved in neurogenesis. At the same time, the number of genes co-expressed with *ND2* or *ND4* collapses in this model. *MYT1L* haploinsufficiency leads to upregulation of genes involved in neurodevelopment accompanied by the decreased expression of genes associated with neuronal maturation [79]. In these mice, *ND3*, which is not co-expressed with any group of genes involved in the same biological process, becomes co-expressed with genes associated with translation and, especially, with translation in synapse, which is also a neuron-specific process. On the other hand, a previous study of mitochondrial respiratory complexes’ subunit co-expression pattern in hepatocytes revealed that co-expression of genes involved in OXPHOS with genes associated with translation appears only in chemically treated cells [80]. Thus, the biological consequences of identified changes need further studies.

The findings of this study should be considered in light of several limitations. First, the analysis was constrained by the availability of relevant datasets in the GEO repository, with only two datasets meeting the inclusion criteria. Additionally, the three rodent models of ADHD used in this study, including *DAT*-KO rats, exhibit complex and heterogeneous phenotypes that extend beyond hyperactivity, potentially introducing variability into the results. Second, the transcriptomic datasets analyzed were derived from narrow study groups across different laboratories, which may limit the generalizability of the findings. Furthermore, while qPCR provided targeted validation of mitochondrial gene expression, this approach has inherent limitations compared to whole-transcriptome methods like RNA-Seq, particularly in terms of discovering broader co-expression networks. Importantly, our qPCR results partially reproduced findings from previous RNA-Seq studies in other ADHD models, supporting the reproducibility of our observations. Although, last but not least, the overlapping coding sequences of *ND4* and *ND4L* may introduce technical artifacts in qPCR measurements, necessitating cautious interpretation of their expression levels.

The overall level of gene expression is influenced by multiple factors on post-transcriptional and post-translational levels. Therefore, comprehensive assessment of gene expression requires integrated approaches at different biological levels. As a result, the functional implications of altered mitochondrial gene mRNA expression in ADHD models warrant further investigation, both at the protein level and in the context of mitochondrial function.

While these factors reduce the reliability of direct comparisons and unified conclusions, they also align with our goal of identifying reproducible changes across different models, which can be considered a strength of the study. Finally, this study did not include a detailed analysis of mitochondrial functional characteristics in *DAT*-KO and *DAT*-Het rat PFC samples, such as ROS production and mitochondrial membrane potential. These measurements could provide vital information about the functional consequences of altered *NDs* co-expression patterns and might reveal latent impairments in mitochondrial function. Addressing these limitations in future studies will provide an opportunity to elucidate the compensatory vs. maladaptive nature of mitochondrial restructuring in ADHD models.

In the present study, we compared three ADHD models, whose phenotypes are very different. *Adgrl3*-KO mice are characterized by impairments across spatial memory and learning domains, increased impulsivity and sociability, and decreased aggression [81]. The *MYT1L*-Het model demonstrates a more severe phenotype with microcephaly, muscle weakness and fatigue, obesity, and social orientation deficits. *DAT*-KO rats are characterized by the impairment of working memory, emotion, response timing, action planning, and attentional control, i.e., disorders related both to ADHD and autistic spectrum disorders. These rats also demonstrate lower body size and weight and changes in posture compared to WT littermates [82]. Our results were generally reproduced in these quite different contexts. Additionally, we revealed similar co-expression enhancements in *DAT*-Het rats compared to WT littermates. These animals do not demonstrate hyperactivity or attention impairment; meanwhile the revealed changes may be considered an ADHD endophenotype in these DA-deficient rats.

As the participants of ADHD pathogenesis, mitochondria are also considered the prospective treatment targets. Antioxidants, which improve mitochondrial activity and energy metabolism, also are studied as adjuvant therapy for ADHD [83,84]. Currently, an ameliorative effect on ADHD symptoms was identified for omega-3 [85] or polyphenols [86,87], which are known antioxidants with an identified effect on mitochondrial function [85,88]. In the present study, we revealed the possible changes in mtDNA gene co-regulation in ADHD models. Future research should focus on elucidating the functional consequences of these co-expression shifts and their impact on mitochondrial bioenergetics and ROS production, as well as evaluating the therapeutic potential of targeting these pathways in ADHD.

## 4. Materials and Methods

### 4.1. Gene Expression Omnibus Database Search

The RNAseq data were mined from the public database GEO from NCBI [36] by the search requests “ADHD”, “Hyperactive”, or “Hyperactivity” in GEO DataSets database. Search results were filtered by method, species, and sample number (*n* = 10). Only datasets generated by “Expression profiling by high-throughput sequencing” of human, mouse or rat samples were selected. The inclusion criteria were as follows: (1) at least 5 PFC samples per study subgroup in the dataset, including intact WT samples; (2) the datasets represent the expression profiles of native samples; data for single-cell RNA sequencing or cell fractions were excluded.

### 4.2. RNA-Seq Data Normalization and Statistical Analysis

For unification of data processing, raw counts for selected datasets were downloaded from the GREIN web platform, which provides data obtained from the GEO SRA files reanalyzed by the Salmon v0.12.0 processing tool [89]. Raw counts were CPM (count per million) normalized by the edge R package (v. 4.6.2). Only CPM values exceeding a threshold of 0.5 were retained for downstream analysis, aligning with cutoff criteria established in the Expression Atlas database [90]. Gene expression data were subsequently visualized using the ggplot2 package (version 3.5.2) in R (v.4.5.0.) [91].

Differential gene expression analysis was conducted using the quasi-likelihood framework implemented in edgeR [92], following preliminary filtering with the filterByExpr function (default parameters). Data normalization was performed using the calcNormFactors function in edgeR, applying the trimmed mean of M-values (TMM) method. To control for multiple comparisons, *p*-values were adjusted using the Benjamini–Hochberg procedure, and genes with adjusted *p*-values (*p*adj) < 0.05 were classified as differentially expressed.

Additionally, Pearson correlation coefficients were calculated to assess relationships between gene expression levels. The results were visualized using the corrplot package (version 0.95) in R [68], with correlations deemed statistically significant at *p* < 0.05.

### 4.3. Measurement of Co-Expression Profiles and Functional Analysis

To identify co-expression partners for each *ND* gene, we calculated Pearson correlation coefficients across the dataset and retained only those genes with statistically significant correlations (*p* < 0.05). From this set, the top 100 most strongly co-expressed genes were selected for further analysis, considering that thiselic size of gene cluster is commonly applied for different kinds of gene-phenotype tests with robust results [93] and allows for comparing trends in data from different sources [94].

We then performed Gene Ontology (GO) enrichment analysis [95], focusing specifically on the “Biological Process” (BP) category, to compare functional annotations across the identified gene clusters. Enrichment analysis was conducted using the compareCluster function in the clusterProfiler [96] package (version 4.16.0), which enabled a cross-cluster comparison of GO BP term enrichment. Only GO BP terms with a false discovery rate (FDR) < 0.05 were considered statistically significant.

To visualize the results of the functional enrichment analysis, we employed the “dotplot” function from the enrichplot package (version 1.28.2), which leverages ggplot2-based graphics for clear and informative representation of the data.

### 4.4. Animals

This study utilized prefrontal cortex tissue samples from adult male rats at five months of age, divided into three distinct groups: homozygous DAT knockout (*DAT*-KO, *n* = 11), heterozygous *DAT* knockout (*DAT*-Het, *n* = 13), and wild-type controls (WT, *n* = 6). The animals were generated through crossbreeding of heterozygous carriers, and their genotypes were confirmed using a standardized genotyping protocol described earlier [37].

Throughout the experiment, rats were housed in controlled environmental conditions within individually ventilated cages (IVC; RAIR IsoSystem World Cage 500, Lab Products, Inc., Seaford, DE, USA). They had unrestricted access to food and water, with environmental parameters maintained at 50–70% humidity, a 12 h light/dark cycle (lights activated at 09:00), and a stable ambient temperature of 22 ± 1.0 °C.

All procedures involving animals were conducted in strict compliance with ethical guidelines and were approved by the Institutional Animal Care and Use Committee of Saint Petersburg State University (protocol no. 131-03-6, approved on 25 April 2025).

For PFC tissue collection, rats were humanely euthanized via decapitation under deep isoflurane anesthesia (1% isoflurane in 100% oxygen, supplied by Chemical Iberica Produktos Veterinarios, Salamanca, Spain). Immediately following euthanasia, PFC samples were excised and preserved in ExtractRNA reagent (Evrogen, Moscow, Russia) to ensure RNA integrity.

### 4.5. RNA Isolation, Reverse Transcription, and Quantitative Polymerase Chain Reaction (qPCR)

For RNA extraction, prefrontal cortex tissue samples were processed using the ExtractRNA reagent (Evrogen, Moscow, Russia), following the protocol provided by the manufacturer. The quantity and purity of the isolated RNA were determined spectrophotometrically with a NanoDrop 2000 spectrophotometer (Thermo Scientific, Waltham, MA, USA).

Subsequently, complementary DNA (cDNA) synthesis was performed using 300 ng of total RNA per sample. Reverse transcription reactions were carried out with the MMLV Reverse Transcriptase kit (Evrogen, Moscow, Russia), adhering to the recommended experimental conditions.

Gene-specific primers utilized in this study are provided in Table 2. Each cDNA sample underwent at least two independent qPCR runs using the CFX96 Touch Real-Time PCR Detection System (Bio-Rad, Hercules, CA, USA). The transcript levels of *ND1–ND6* were quantified using qPCRmix-HS SYBR (Evrogen, Moscow, Russia). To evaluate gene expression levels, quantitative polymerase chain reaction (qPCR) was conducted by 40 thermal cycles, which include 10 s at 95 °C, 15 s at 60 °C, and 30 s at 72 °C, followed by fluorescence acquisition. To confirm amplicon specificity, a melting curve analysis was performed for each amplification product. The melting curve was generated by applying the default CFX96 Touch Real-Time PCR Detection System (Bio-Rad, Hercules, CA, USA) protocol.

### 4.6. ATP Measurements

PFC samples immediately upon excision were flash-frozen in liquid nitrogen and stored at −80 °C until needed. On the day of the assay, samples were homogenized in a tissue ball mill (Retsch MM 400) (Retsch, Haan, Germany) for 5 min at 30 Hz with stainless steel balls (Quiagen Metal Beads 2.38 mm, (Qiagen, Frederick, MD, USA) in 50 mM Tris-HCl, 150 mM NaCl, 5 mM EDTA solution; pH 7.3–7.5, heated to 90–95 °C. The resultant crude homogenate was immediately cooled on ice and centrifuged (at 10,000× *g* at 4–5 °C for 10 min), and clear supernate was used for determining ATP concentration immediately after. The ATP levels were measured using the CellTiter-Glo^®^ Luminescent Cell Viability Assay kit (Promega, Fitchburg, WI, USA). This assay system, through an ATP-dependent luciferin-luciferase reaction, produces light, whose intensity is proportional to the sample’s ATP content. 100 mkL of clarified homogenate (see above) were mixed with an equivalent volume of CellTiter-Glo^®^ (Promega, WI, USA) working reagent in a white opaque 96-well plate (Corning Incorporated, Corning, NY, USA). Blank wells contained 100 mcL of 50 mM Tris-HCl, 150 mM NaCl, and 5 mM EDTA solution (see above) mixed with an equivalent volume of CellTiter-Glo^®^ working reagent. The suspension was shaken for 2–3 min and incubated at room temperature for 10 min in the dark. Luminescence was then quantified on the Mithras LB940 (Berthold Technologies, Bad Wildbad, Germany) multimodal plate reader. Dilutions of Sodium-ATP solution Trifosalenin, 10 mg/mL, (Ellara, Saint-Petersburg, Russia) 10 mg/mL (~16.5 mM) was applied as a standard, and a calibration curve was constructed at a range of 5 μM–0.1 nM ATP.

### 4.7. Statistical Analysis

To quantify relative gene expression levels, we employed the 2^−ΔΔCt^ method. The analysis involved several sequential steps: first, the cycle threshold (Ct) values were recorded for each sample. Next, the ΔCt values were calculated by subtracting the Ct of the housekeeping gene (*Gapdh*) from the Ct of the target gene. The mean Ct value for each gene in the WT group was then subtracted from the ΔCt values to obtain ΔΔCt. Finally, the 2^−ΔΔCt^ values were computed to determine relative expression levels.

Each assay was conducted in duplicate, and mean values were used for subsequent statistical analysis. Prior to comparisons, data were assessed for normal distribution using the Shapiro–Wilk test. Differences in normalized expression levels were evaluated with either a Student’s *t*-test (for normally distributed data, where *p* > 0.05 across all groups) or a Mann–Whitney U test with Holm–Bonferroni correction for multiple comparisons. Outliers were detected using the interquartile range (IQR) criterion and excluded from further analysis.

For ATP level comparisons between groups, the Mann–Whitney U test was applied. To account for multiple testing, *p*-values were adjusted using the Benjamini–Hochberg procedure. Statistical significance was defined as adjusted *p*-values (*p*_adj_) < 0.05.

Data visualization was performed using the ggplot2 package (version 3.6.2) in the R programming environment [91].

Pearson’s correlation between genes’ ΔCt values was calculated and visualized with the corrplot (v. 0.95) R package [97]. Correlations were considered significant if *p*-values were less than 0.05.

## 5. Conclusions

ADHD is currently recognized as a neurodevelopmental condition characterized by impairments in attention and/or impulsivity, representing one of several disorders within a broader clinical spectrum [98]. In this study, we compared the mRNA expression of seven mitochondrial DNA-encoded electron transport chain complex I subunits (ND1–ND6) in the PFC across three distinct ADHD animal models: *MYT1L*-Het mice, *Adgrl3*-KO mice, and *DAT*-KO rats.

Our analysis of available transcriptomic data revealed consistent profiles of *ND1–ND6* gene expression in the PFC of mice. While the expression levels of individual *ND* genes remained stable across all three ADHD models, we observed an increase in co-expression among these genes. This pattern was particularly pronounced in *DAT*-Het rats, which do not exhibit hyperactivity, yet their co-expression profile differed markedly from that of their *DAT*-KO littermates.

Despite these shifts in co-expression, measurements of ATP concentrations in PFC samples from *DAT*-Het and *DAT*-KO rats did not reveal significant changes. This suggests that the functional implications of altered *ND* gene co-expression—including potential effects on ROS production or compensatory mechanisms—require further investigation. Additionally, we identified shifts in the co-expression patterns between *ND* mRNA and other genes in mouse ADHD models. Notably, *ND3* and *ND4L* demonstrated co-expression with genes associated with neural system functioning. Future studies should focus on elucidating the molecular mechanisms underlying these co-expression shifts and their potential role in ADHD pathophysiology.

## Figures and Tables

**Table 1:** RNAseq datasets included in the analysis.

Dataset ID	Title	Sequencing Platform	Samples Characteristics	Additional Information
GSE173926	A MYT1L Syndrome mouse model recapitulates patient phenotypes and reveals altered brain development due to disrupted neuronal maturation [RNA-Seq]	Illumina NovaSeq 6000	PFC, adult male and female samples, heterozygous *MYT1L* mutants (*n* = 6) or wild-type (*n* = 6) mice	Heterozygous *MYT1L* mutants are hyperactive across numerous tasks, including open field, social operant and PPI/startle, as well as altered sociality [41].
GSE117357	Differential Gene Expression in *Adgrl3* KO Mice	Illumina NovaSeq 500	PFC, adult samples, *Adgrl3* KO (*n* = 10) or wild-type (*n* = 10) mice	*Adgrl3*-deficient mice demonstrate increased locomotive activity, impulsivity, decreased visuospatial and recognition memory, and sociability [42].

**Table 2:** Primers and probes were used for quantitative RT-PCR in *DAT*-KO and DAT-Het rat study.

Gene	Forward Primer	Reverse Primer
*ND1*	CAA CTA CGC AAA GGC CCC AAC A	GGG AGA GGG TTG GGG CGA TAA T
*ND2*	GGA CTA GCC CCC TTC CAC TA	GGC GCC AAC AAA GAC TGA TG
*ND3*	CCC AAC AAG TTC TGC ACG CCT T	TTG AAT CGC TCA TGG GAG GGG G
*ND4*	CCC ACG GCT TAA CCT CCT CAC T	GGG TGG TAG TGC TAG GTT GGC T
*ND4L*	ACT CTC CTC TGC CTA GAA GGA A	AAA CCT ACT GCT GCT TCG CA
*ND5*	CGG CCC TCC AAG CAA TCC TCT A	GGA TGA AGT CCG AAT TGG GCG G
*ND6*	ACT GGT TGT CTA GGG TTG GCG T	CCC TCA AGT CTC CGG GTA CTC C
*Gapdh*	CGC CTG GAG AAA CCT GCC AAG	CTG GTC CTC AGT GTA GCC CAG G

## Data Availability

The original contributions presented in this study are included in the article/Supplementary Materials. Further inquiries can be directed to the corresponding author.

## Supplementary Materials

The following supporting information can be downloaded at: https://www.mdpi.com/article/10.3390/ijms262211079/s1.
