# Supplementary material for: Altered Co-Expression Patterns of Mitochondrial NADH-Dehydrogenase Genes in the Prefrontal Cortex of Rodent ADHD Models
Source: Int J Mol Sci. 2025 Nov 16;26(22):11079. doi: 10.3390/ijms262211079 (PMC12652654; doi:10.3390/ijms262211079)
Supplement: Supplementary file 1 [file ijms-26-11079-s001.zip › SUPP/Supp_3.pdf]

Figure S1. GO BP term enrichment for the top 100 genes co-expressed with NDs in the PFC in control WT mice in GSE117357 (A) and GSE173926 (B), Adgrl3-KO mice in the GSE117357 dataset (C), and *MYT1L* heterozygous knockout (GSE173926, D). The number below the gene name represents the number of genes involved in the analysis after the transformation of Ensembl gene IDs to Entrez IDs.

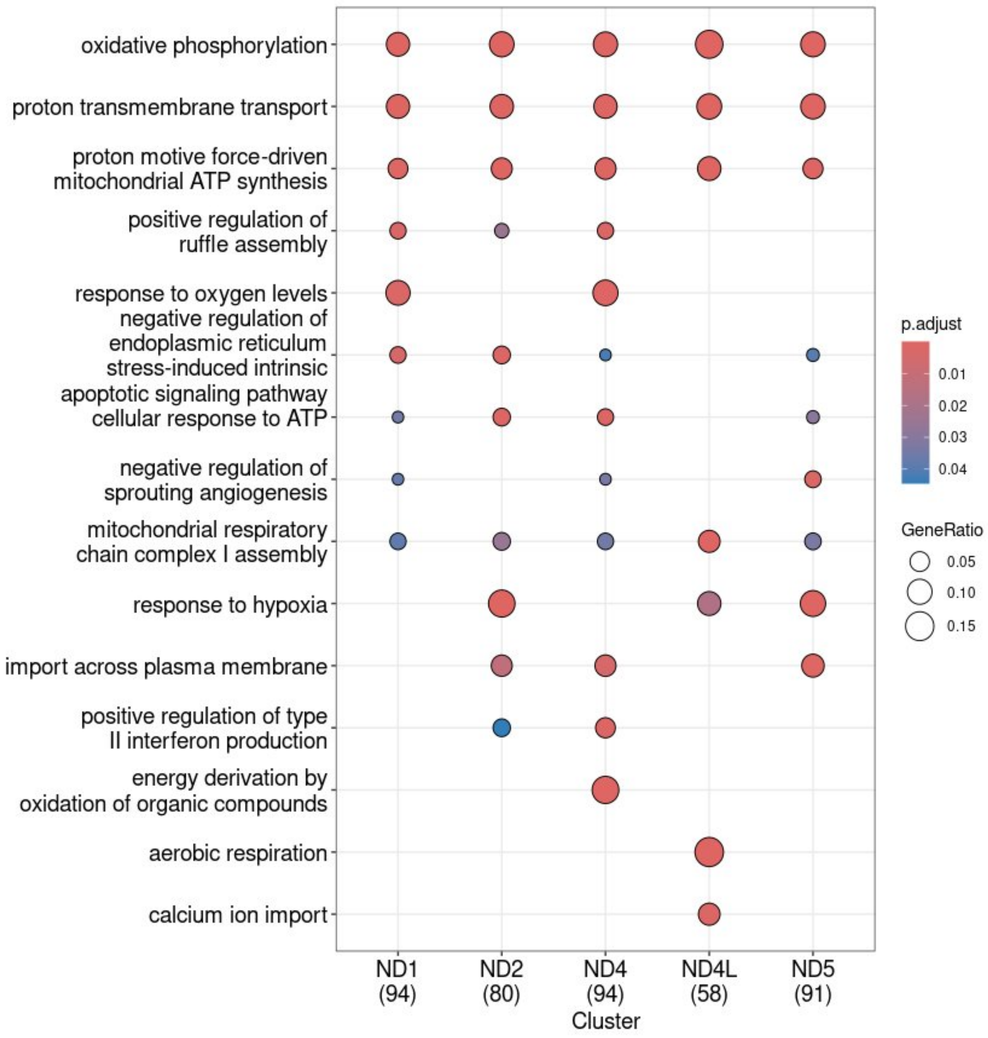

A

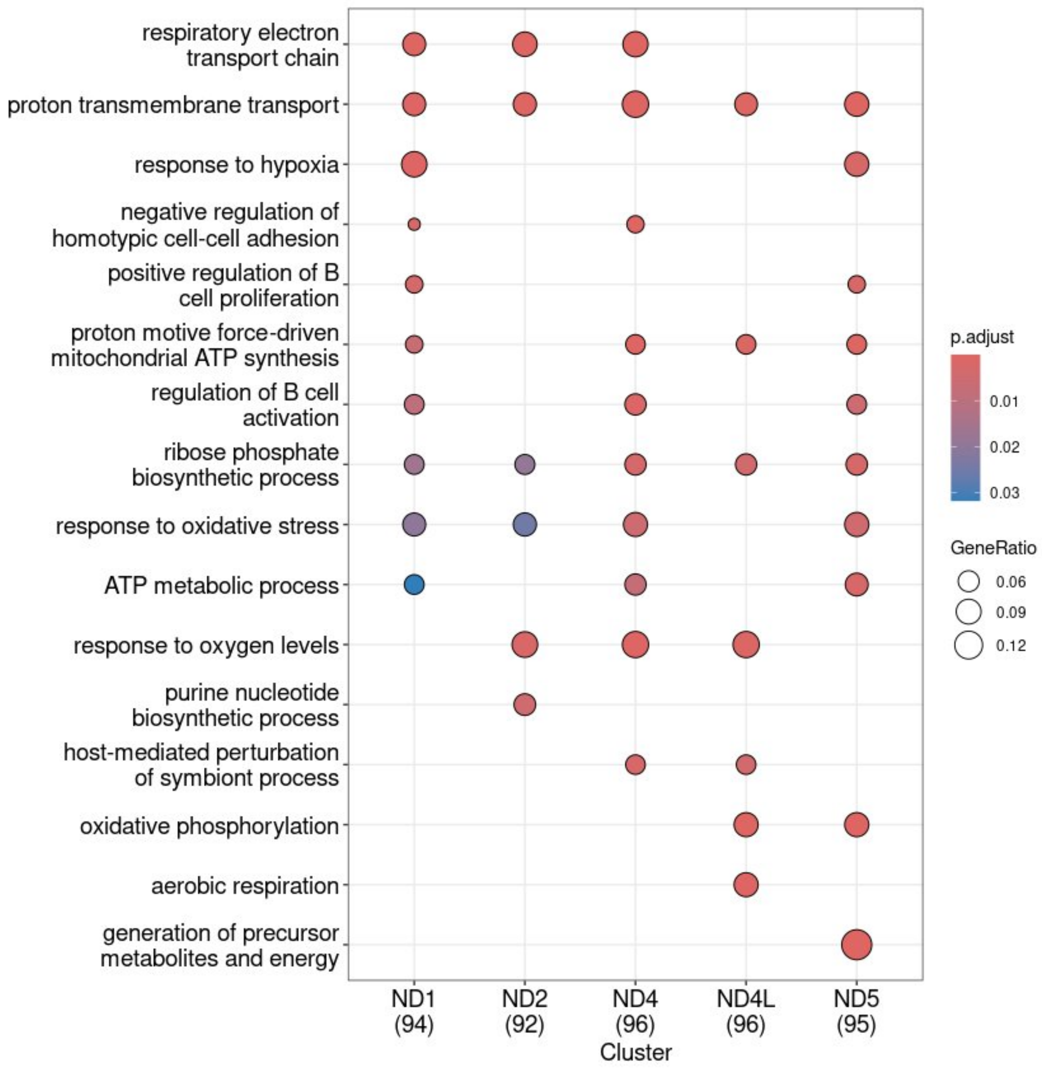

B

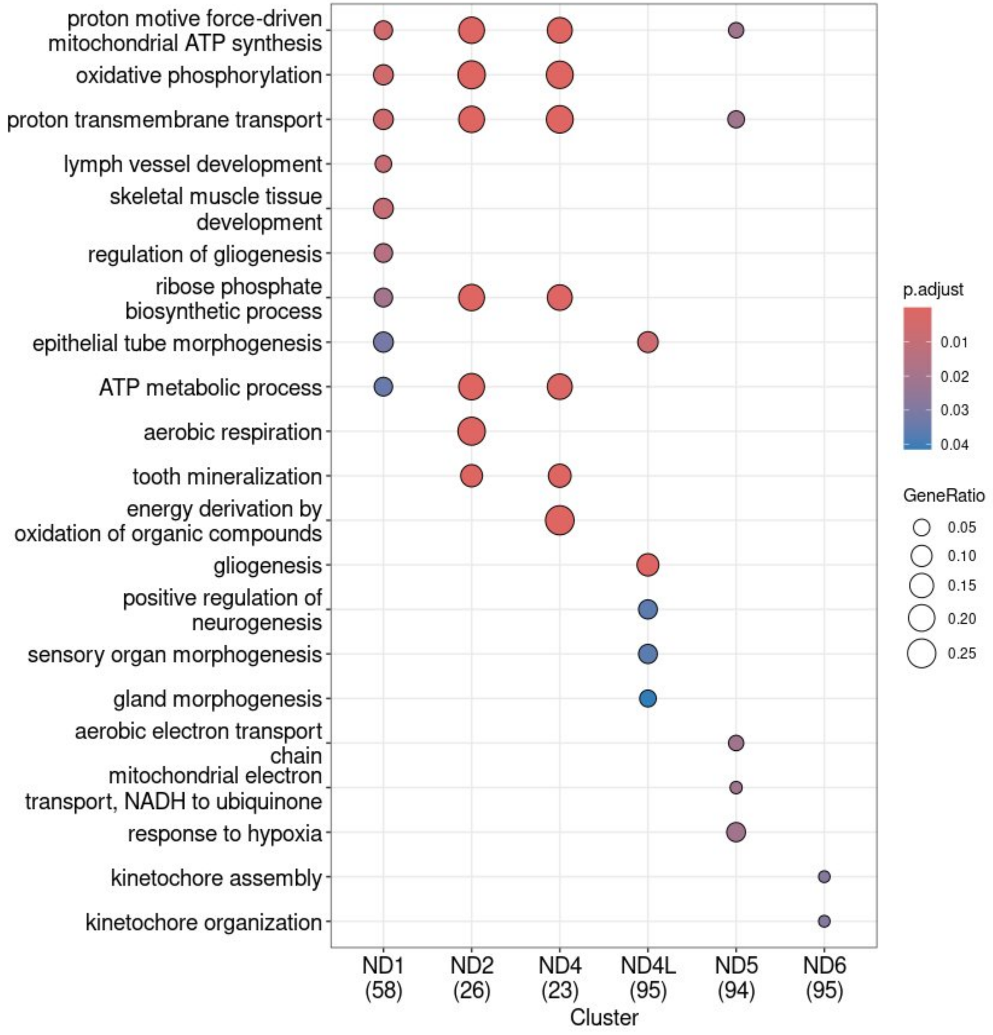
proton motive force-driven mitochondrial ATP synthesis
oxidative phosphorylation
proton transmembrane transport
lymph vessel development
skeletal muscle tissue development
regulation of gliogenesis
ribose phosphate biosynthetic process
epithelial tube morphogenesis
ATP metabolic process
aerobic respiration
tooth mineralization
energy derivation by oxidation of organic compounds
gliogenesis
positive regulation of neurogenesis
sensory organ morphogenesis
gland morphogenesis
aerobic electron transport chain
mitochondrial electron transport, NADH to ubiquinone
response to hypoxia
kinetochore assembly
kinetochore organization
ND1 (58)
ND2 (26)
ND4 (23)
ND4L (95)
ND5 (94)
ND6 (95)
Cluster
p.adjust
0.01
0.02
0.03
0.04
GeneRatio
0.05
0.10
0.15
0.20
0.25

C

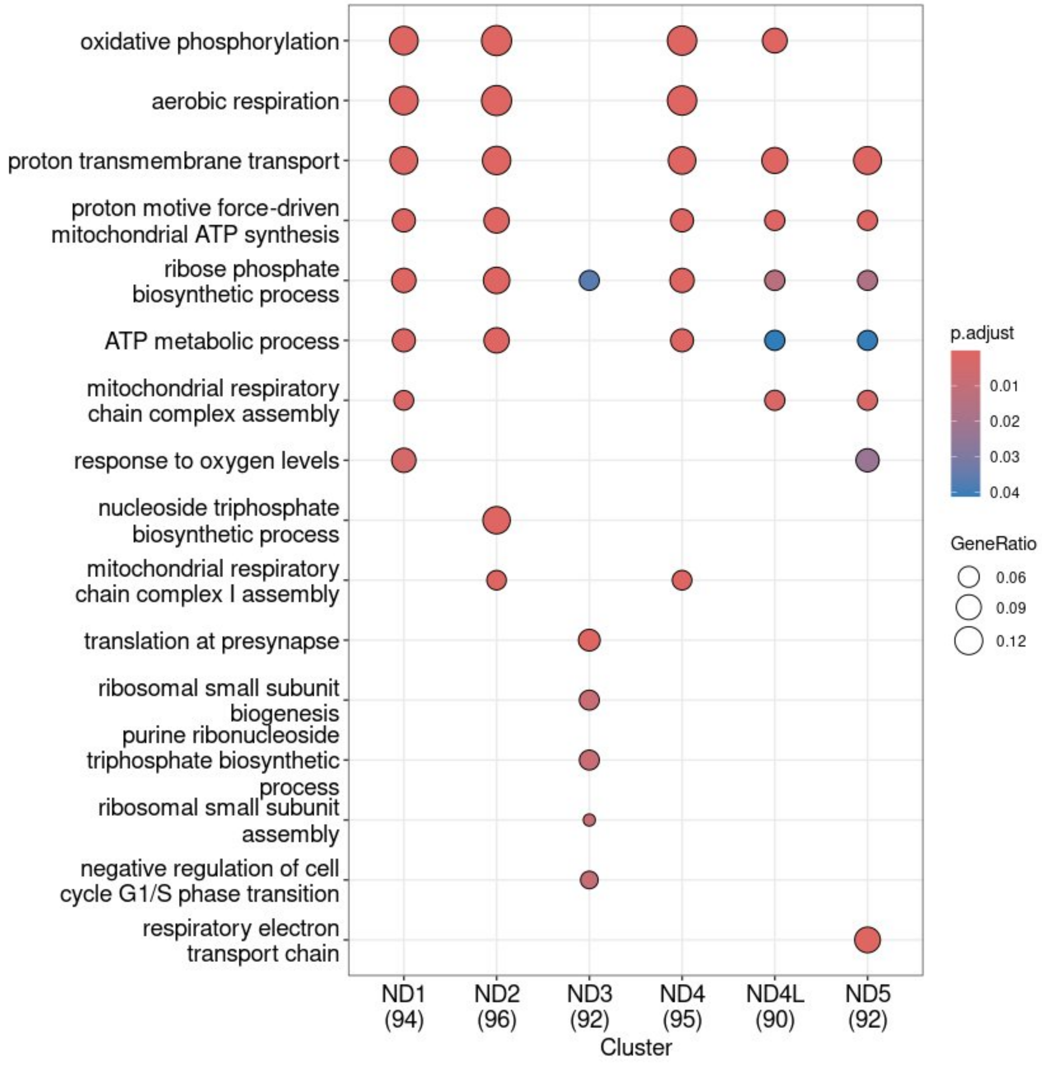
oxidative phosphorylation
aerobic respiration
proton transmembrane transport
proton motive force-driven mitochondrial ATP synthesis
ribose phosphate biosynthetic process
ATP metabolic process
mitochondrial respiratory chain complex assembly
response to oxygen levels
nucleoside triphosphate biosynthetic process
mitochondrial respiratory chain complex I assembly
translation at presynapse
ribosomal small subunit biogenesis
purine ribonucleoside triphosphate biosynthetic process
ribosomal small subunit assembly
negative regulation of cell cycle G1/S phase transition
respiratory electron transport chain
ND1 (94)
ND2 (96)
ND3 (92)
ND4 (95)
ND4L (90)
ND5 (92)
Cluster
p.adjust
0.01
0.02
0.03
0.04
GeneRatio
0.06
0.09
0.12

D
